# Clinical and biological heterogeneity of multisystem inflammatory syndrome in adults following SARS-CoV-2 infection: a case series

**DOI:** 10.3389/fmed.2023.1187420

**Published:** 2023-07-06

**Authors:** Kaia E. Barth, Natasha Spottiswoode, Charlotte Hurabielle, Lakshmi Subbaraj, COMET Consortium, Carolyn S. Calfee, Michael A. Matthay, Sarah French, Andrew Connolly, Stephen M. Hewitt, Kevin M. Vannella, Christopher Barnett, Charles R. Langelier, Sarah Patterson

**Affiliations:** ^1^Department of Medicine, University of California, San Francisco, San Francisco, CA, United States; ^2^Division of Infectious Diseases, University of California, San Francisco, San Francisco, CA, United States; ^3^Division of Rheumatology, University of California, San Francisco, San Francisco, CA, United States; ^4^Division of Pulmonary, Critical Care, Allergy, and Sleep Medicine, University of California, San Francisco, San Francisco, CA, United States; ^5^Department of Pathology, University of California, San Francisco, San Francisco, CA, United States; ^6^Laboratory of Pathology, Center for Cancer Research, National Cancer Institute, National Institutes of Health, Bethesda, MD, United States; ^7^Emerging Pathogens Section, Critical Care Medicine Department, Clinical Center, National Institutes of Health, Bethesda, MD, United States; ^8^Laboratory of Virology, National Institute of Allergy and Infectious Diseases, Bethesda, MD, United States; ^9^Division of Cardiology, University of California, San Francisco, San Francisco, CA, United States; ^10^Chan Zuckerberg Biohub, San Francisco, CA, United States

**Keywords:** COVID-19, MIS-A, myocarditis, SARS-CoV-2, multisystem inflammatory syndrome, DKA

## Abstract

**Importance:**

Multisystem inflammatory syndrome in adults (MIS-A) is a poorly understood complication of SARS-CoV-2 infection with significant morbidity and mortality.

**Objective:**

Identify clinical, immunological, and histopathologic features of MIS-A to improve understanding of the pathophysiology and approach to treatment.

**Design:**

Three cases of MIS-A following SARS-CoV-2 infection were clinically identified between October 2021 – March 2022 using the U.S. Centers for Disease Control and Prevention diagnostic criteria. Clinical, laboratory, imaging, and tissue data were assessed.

**Findings:**

All three patients developed acute onset cardiogenic shock and demonstrated elevated inflammatory biomarkers at the time of hospital admission that resolved over time. One case co-occurred with new onset Type 1 diabetes and sepsis. Retrospective analysis of myocardial tissue from one case identified SARS-CoV-2 RNA. All three patients fully recovered with standard of care interventions plus immunomodulatory therapy that included intravenous immunoglobulin, corticosteroids, and in two cases, anakinra.

**Conclusion:**

MIS-A is a severe post-acute sequela of COVID-19 characterized by systemic elevation of inflammatory biomarkers. In this series of three cases, we find that although clinical courses and co-existent diseases vary, even severe presentations have potential for full recovery with prompt recognition and treatment. In addition to cardiogenic shock, glucose intolerance, unmasking of autoimmune disease, and sepsis can be features of MIS-A, and SARS-CoV-2 myocarditis can lead to a similar clinical syndrome.

## Introduction

Early in the COVID-19 pandemic, reports emerged of a new multisystem inflammatory syndrome in children (MIS-C) following SARS-CoV-2 infection (1, 2). A similar syndrome, termed MIS-A, was later described in adults (3, 4). The United States Centers for Disease Control and Prevention defines MIS-A as the presence of fever and at least one primary criterion (severe cardiac disease, rash with conjunctivitis), two secondary criteria (neurological symptoms, shock not attributable to other cause, abdominal pain, vomiting or diarrhea, and thrombocytopenia), and laboratory criteria including evidence of prior SARS-COV-2 infection and at least two markers of inflammation [elevated erythrocyte sedimentation rate (ESR), C-reactive protein (CRP), ferritin, procalcitonin, interlukin-6 (IL-6)] in the absence of an alternative diagnosis in adults age 21 and older (3, 5). Several immunologic mechanisms underlying MIS-A have been proposed, however the pathophysiology remains poorly understood (3).

Here, we present three cases of presumed MIS-A along with biochemical and histologic data which together enhance our understanding of the heterogenous pathophysiology, clinical course, and appropriate treatment of this life-threatening disease.

## Methods

We identified patients admitted to the University of California San Francisco (UCSF) hospitals meeting the CDC case definition for MIS-A between September 2021 and March 2022 by querying faculty within the Divisions of Infectious Diseases and Rheumatology. One patient (Case 1) was enrolled in the prospective, observational, COVID-19 Multi-phenotyping for Effective Therapies (COMET) study under UCSF institutional review board protocol 20-30497. Verbal informed consent was obtained from all patients for their anonymized information to be published in this article. Plasma cytokines were measured by multiplex enzyme linked immunosorbent assay (ELISA) from Case 1 at four timepoints, as well as from 20 convalescent control patients sampled 3 months post COVID-19 diagnosis. Myocardial tissue from Case 3 was sent to the National Institutes of Health (NIH) for additional immunology and histopathology testing.

## Results

**Figure 1:**
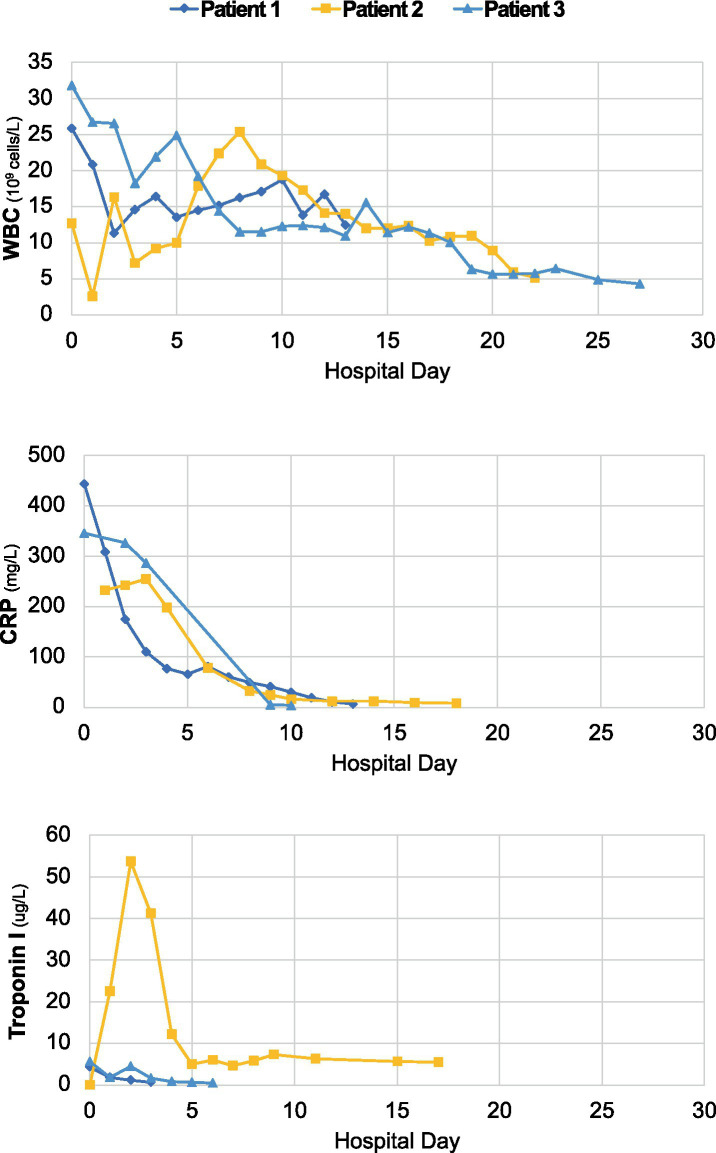
Longitudinal dynamics of white blood cell count (WBC), C-reactive protein (CRP) and troponin-I over the course of hospitalization for each case.

We identified 3 cases that met inclusion criteria, each of which presented 10 days to 8 weeks after a proven or probable (compatible clinical history and positive SARS-CoV-2 nucleocapsid IgG) mild, self-limiting SARS-CoV-2 infection. All cases involved young men who developed acute heart failure and cardiogenic shock, two of whom required mechanical circulatory support. We describe the clinical course for each (Table 1), and their associated imaging and laboratory data (Figure 1; Supplementary Table S1).

**Table 1:** Clinical and laboratory characteristics.

Case	Age/Sex	COVID-19 diagnosis	COVID-19 vaccination status	Clinical presentation	Notable laboratory data	Notable cardiac data	Treatments received	Outcomes	Hospital length of stay
1	34-year-old male	Clinical syndrome consistent with COVID-19 two months prior to admission; negative SARS-CoV-2 PCR; Positive SARS-CoV-2 nucleocapsid IgG on hospital day 1	Not vaccinated	Febrile respiratory illness 2 months prior to admission. Presented with worsening dyspnea, found to be in cardiogenic shock.	Troponin 4.24 μg/L; BNP 1618 pg./mL; CRP 443 mg/L; WBC 25.9×10^9^/L; Ferritin 5,056 μg/L; blood glucose 382 mg/dL	TTE: global hypokinesis; LVEF: 20–25%; moderately decreased RVEF; no valvular disease. No cardiac catheterization performed.	Pulse dose steroids followed by prednisone taper, 2-day course IVIG, anakinra, vasopressors, dobutamine, LVAD	LVEF normalized, discharged home	14 days
2	28-year-old male	Positive SARS-CoV-2 PCR (Ct 36.1) hospital day 1, positive SARS-CoV-2 nucleocapsid IgG hospital day 2	Not vaccinated	1-week prodrome of fatigue, polydipsia, polyuria progressing to severe delirium. Found to be in diabetic ketoacidosis and hypovolemic, cardiogenic, and septic shock.	Troponin 50 μg/L; BNP 300 pg./mL; CRP 232 mg/L, ferritin 15,000 μg/L; Blood glucose 1,959 mg/dL, hemoglobin A1c > 14%, pH < 6.78	TTE: global hypokinesis; LVEF 15%; moderately decreased RVEF; no valvular disease. No coronary artery disease.	Pulse dose steroids followed by prednisone taper, 2-day course IVIG, anakinra, antibiotics, remdesivir, vasopressors, dobutamine, insulin, LVAD, VA-ECMO, mechanical ventilation, renal replacement therapy	LVEF normalized, discharged to acute rehab with ongoing need for hemodialysis and insulin	24 days
3	31-year-old male	Positive SARS-CoV-2 PCR (Ct 34.2) hospital day 4; positive SARS-CoV-2 nucleocapsid IgG hospital day 5	First dose of Moderna COVID-19 vaccine given 5 weeks prior to admission	10-day prodromal illness of fevers, pharyngitis, and abdominal pain. Presented with acute dyspnea in cardiogenic shock complicated by cardiac arrest.	Troponin 4.55 μg/L;NT-Pro-BNP 34,645 pg./dL; CRP 362.7 mg/L; WBC 31.8×10^9^/L; Ferritin 8,219 μg/L	Global hypokinesis, LVEF 25%; moderate-severely decreased RVEF; mild tricuspid valve insufficiency. No coronary artery disease. SARS-CoV-2 RNA present on cardiac biopsy	Pulse dose steroids followed by prednisone taper, 5-day course IVIG, antibiotics vasopressors, dobutamine, mechanical ventilation	LVEF normalized, discharged to acute rehab with residual L sided deficits due to stroke	35 days

BNP, brain natriuretic peptide; CRP, C-reactive protein; WBC, white blood cell count; TTE, transthoracic echocardiogram; LVEF, left ventricular ejection fraction; RVEF, right ventricular ejection fraction; Ct, cycle threshold; LVAD, left ventricular assist device.

### Case 1

In February 2022, a 34-year-old male with a history of obstructive sleep apnea, anxiety and degenerative disc disease presented with one week of worsening dyspnea, cough, fevers, diarrhea, conjunctivitis, and rash, two months after an upper respiratory syndrome associated with loss of taste and smell, during which he was not tested for COVID-19. His demographic data includes Hispanic ethnicity, body mass index (BMI) of 22, not vaccinated against COVID-19, not taking any medications. His temperature was 38.8°C, blood pressure 83/61 mmHg, heart rate 130 beats per minute (bpm), respiratory rate (RR) 37 breaths per minute, and he required 2 liters per minute of supplemental oxygen.

His laboratory findings included a white blood cell (WBC) count of 25.9×10^9^/L (3.4–10.0 x10E9/L), platelet count of 87,000/μL (140–450 x10E9/L), hemoglobin of 4.8 g/dL (13.6–17.5 g/dL), and elevated inflammatory markers including a CRP of 443 mg/L (<5.1 mg/L), ESR of 73 mm/h (0–10 mm/h), IL-6 of 27.8 pg./dL (<5.00 pg./mL), and ferritin of 5,056 μg/L (48–420 μg/L). He had a troponin-I of 4.24 μg/L (<0.02), brain natriuretic protein (BNP) of 1,618 pg./mL (<25 pg./mL), and a transthoracic echocardiogram (TTE) demonstrated global hypokinesis with a left ventricular ejection fraction (LVEF) of 20%. He also had acute kidney injury with a creatinine of 2.08 mg/dL (0.73–1.24 mg/dL), elevated from a baseline of 0.9 mg/dL and elevated liver enzymes.

The differential diagnosis for his syndrome included COVID-19, viral myocarditis, acute myocardial infarction (MI), adult-onset Still’s disease, and Kawasaki disease. A SARS-CoV-2 PCR returned negative, but a nucleocapsid IgG assay was positive. Testing for other respiratory viruses including influenza, RSV, parainfluenza, enterovirus/coxsackievirus, parvovirus B19, cytomegalovirus, Epstein–Barr virus and adenovirus returned negative. His electrocardiogram (EKG) was not consistent with acute MI.

He was treated with vasopressors and inotropes including norepinephrine, milrinone and dobutamine, and a percutaneous left ventricular assist device (LVAD) for cardiogenic support. He met the CDC criteria for MIS-A based on his clinical features (age, hospitalization, fever, severe cardiac illness, rash, conjunctivitis, shock, diarrhea) and laboratory findings (thrombocytopenia, elevated CRP, ESR, ferritin, IL-6, and positive SARS-CoV-2 IgG). He was treated with methylprednisolone 1 g daily for 3 days, intravenous immune globulin (IVIG) 2 g/kg daily for 2 days, and anakinra 100 mg twice daily for 5 days followed by once daily for 2 days. No COVID-19 specific treatment was given.

**Figure 2:**
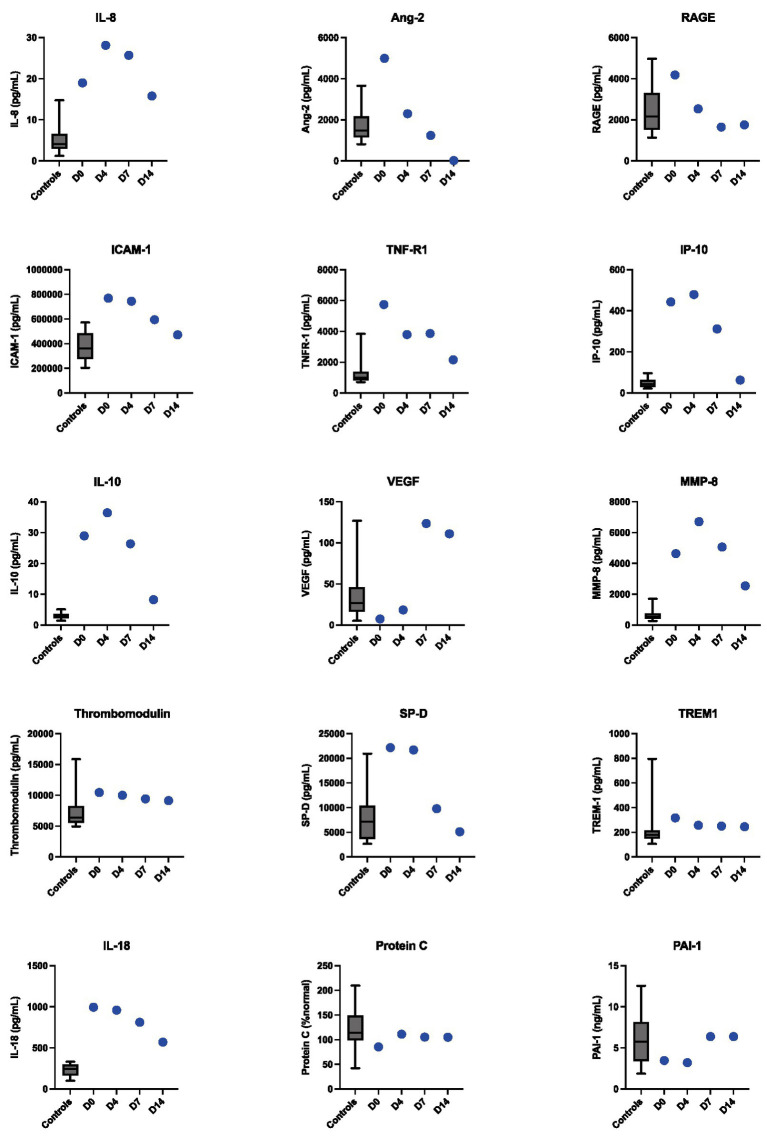
Longitudinal dynamics of inflammatory protein biomarkers levels measured from plasma in Case 1 at hospital days 0, 4, 7, and 14. The box plots represent control values from convalescent plasma samples collected from non-MIS-A control patients 3 months following COVID-19 diagnosis.

To characterize this patient’s dysregulated immune response more deeply, 15 additional inflammatory protein biomarkers were measured from plasma. Compared to post-COVID-19 convalescent controls (Supplementary Table S2), we observed elevations in IL-8, IL-10, IP-10, IL-18, TNFR-1, Ang2, ICAM-1, SP-D, and MMP-8, which peaked by hospital day 7 and then normalized (Figure 2). He demonstrated marked clinical improvement over the course of immunomodulatory treatment. Inotropes/vasopressors were discontinued on hospital day 9, the LVAD was removed on day 10, and a follow-up TTE showed normalization of his LVEF on day 12. He was discharged on day 14 on a prednisone taper. Two weeks following hospital discharge he had recovered normal exercise tolerance and his inflammatory markers had normalized.

### Case 2

In March 2022, a 28-year-old male with no past medical history was found unresponsive on the floor of his home, last seen 15 h prior, following a one-week prodrome of fatigue and polydipsia. His demographic data included non-Hispanic/Caucasian ethnicity/race, BMI of 22, not vaccinated against COVID-19, and no prior medications. On admission, his temperature was 30.0°C, but within 24 h it had risen to 39.0°C. His admission blood pressure was 70/40 mmHg, and he was emergently intubated for airway protection secondary to profound encephalopathy. His initial laboratory findings included pH < 6.78 (7.35–7.45), bicarbonate <5 mEq/L (22–29 mmol/L), creatinine 3.69 mg/dL, blood glucose 1,959 mg/dL (70–199 mg/dL), and lactate 4.8 mmol/L (0.5–2.0 mmol/L). His inflammatory markers were elevated with a CRP of 232 mg/L, ferritin of 15,000 μg/L, and a procalcitonin of 22 μg/L (<0.26 μg/L). A computed tomography (CT) scan revealed diffuse cerebral edema without herniation or hemorrhage, and edematous pancreatitis. A SARS-CoV-2 PCR was positive with a cycle threshold of 36.1, and a nucleocapsid IgG was also positive. The differential diagnosis for his syndrome considered by treating clinicians included sepsis, COVID-19, diabetic ketoacidosis, overdose, toxin exposure, pancreatitis, and myocardial infarction.

The patient underwent volume and metabolic resuscitation to treat severe diabetic ketoacidosis (DKA) and acute pancreatitis, but nonetheless developed progressive shock requiring rapid escalation of vasopressor support. On hospital day 1, he developed emesis, a petechial rash, and a new junctional cardiac rhythm. ECG revealed diffuse ST-segment elevation. His troponin I peaked at 50 μg/L. A TTE showed a left ventricular ejection fraction of 15% with global hypokinesis. On hospital day 2, dobutamine was started, a percutaneous LVAD was placed, and continuous renal replacement therapy was initiated. Blood cultures grew *Staphylococcus aureus* and *Serratia marcescens*. On hospital day 3, veno-arterial extracorporeal membrane oxygenation (VA-ECMO) was initiated.

While on admission the patient was hypothermic, he met clinical and laboratory criteria for MIS-A on hospital day 1 based on the presence of fever, severe cardiac illness, encephalopathy, shock, elevated CRP, ferritin, procalcitonin, and positive SARS-CoV-2 IgG. In addition to antibiotics, fluids, electrolytes and insulin, the patient was treated with methylprednisolone 1 gram IV for 3 days, IVIG 2 g/kg over 2 days, and anakinra 100 mg twice daily for 2 days, then daily for 3 days, and clinically improved. COVID-19 was treated with Remdesivir 100 mg IV daily for 4 days. VA-ECMO and the LVAD was removed on day 8, he was extubated on day 12, and vasopressor and inotropic support was discontinued on day 13. He required ongoing renal replacement therapy for volume removal and was discharged to a skilled nursing facility on hospital day 23 on a prednisone taper and intermittent hemodialysis. His hemoglobin A1c was greater than 14% and testing for islet cell autoantibodies revealed positive GAD-65, Zinc transporter 8, and islet cell antigen152 antibodies, consistent with a new diagnosis of Type 1 diabetes.

### Case 3

In October 2021, a previously healthy 31-year-old man presented with acute dyspnea following a 10-day prodrome of pharyngitis and lymphadenopathy, followed by fever, nausea, vomiting and myalgias for which he had been prescribed antibiotics the day prior. His demographic data includes Hispanic ethnicity, BMI of 32, no prior medications, and he had received an initial dose of SARS-CoV-2 mRNA vaccine 6 weeks prior. On presentation, his temperature was 38.6°C, heart rate 123 bpm, and blood pressure 95/49 mmHg. His initial laboratory assessment was notable for WBC 31.8×10^9^/L, CRP 362.7 mg/L, ESR 90 mm/h, ferritin 8,219 μg/L, creatinine 1.63 mg/dL, troponin 5,713 ng/L (≤45 ng/L), and NT-pro-BNP of 34,645 pg./mL (<125 pg./mL). The differential diagnosis for his syndrome considered by treating clinicians included COVID-19, sepsis, other acute viral illnesses including new-onset HIV, infectious myocarditis due to viral or bacterial pathogens, or autoimmune myocarditis due to giant cell arteritis, sarcoid or systemic lupus erythematosus.

**Figure 3:**
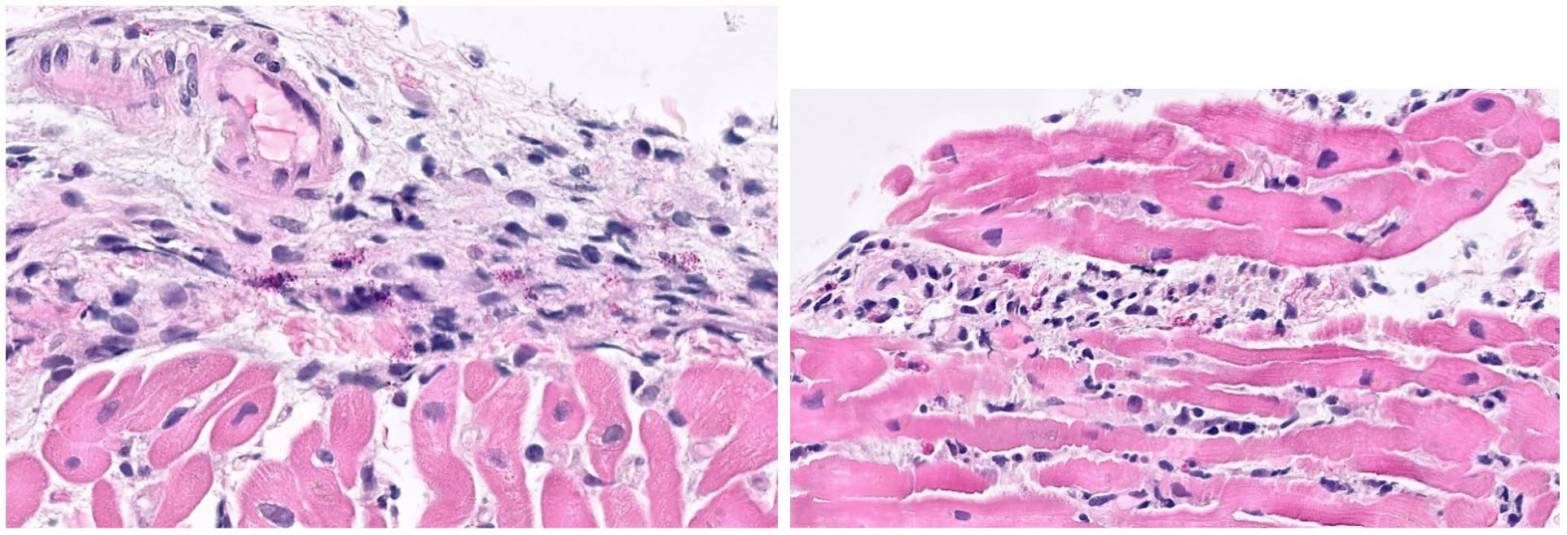
Histological images from endomyocardial biopsy of Case 3. Sections show acute myocarditis with focal myocyte damage associated with mixed inflammatory cell infiltrates containing abundant eosinophils and histiocytes along with a mixture of lymphocytes, plasma cells and occasional neutrophils. The inflammation is particularly prominent in a perivascular and endocardial pattern with extension into the interstitial space.

On hospital day 2, he experienced cardiac arrest with an underlying rhythm of ventricular tachycardia. Following resuscitation, a TTE revealed a LVEF of 25% and global hypokinesis. He required inotropic and vasopressor support with epinephrine, norepinephrine, and dobutamine until hospital day 16. Multiple SARS-CoV-2 PCR tests were performed, only one of which returned positive with a high cycle threshold (34.2). SARS-CoV-2 nucleocapsid IgG antibody was positive. Both blood cultures and tests for other respiratory viral pathogens were negative. An extensive in-hospital workup for infectious (viral and bacterial) and autoimmune myocarditis was unrevealing (Supplementary Table S1). Histopathology from a myocardial biopsy suggested acute myocarditis (Figure 3).

During the patient’s hospitalization, he met CDC criteria for MIS-A (5) based on his clinical features (severe cardiac illness, hypotension, and vomiting) and laboratory findings (positive SARS-CoV-2 serology, elevated CRP, ESR, ferritin, and procalcitonin). He was treated for presumed MIS-A with 5 days of IVIG and 3 days of methylprednisolone 1 g IV daily, followed by a steroid taper. He did not receive anakinra or any targeted COVID-19 treatment. He was also treated empirically with a 7-day course of piperacillin-tazobactam, doxycycline, and acyclovir. His LVEF ultimately normalized on hospital day 25 and he was discharged on hospital day 30. After discharge, his myocardial tissue was sent to the US National Institutes of Health for further testing. SARS-CoV-2 RNA was identified in his myocardial tissue by *in situ* hybridization, consistent with COVID-19 related myocarditis.

## Discussion

Despite the growing number of documented MIS-A cases following COVID-19, the pathophysiology and optimal treatment approach for this syndrome remain incompletely understood. Rapid onset of severe cardiac failure was a key clinical feature in each of the three cases that we describe. In a recent systematic review of 79 MIS-A cases (6), systemic cardiovascular involvement occurred in 81% of cases. Fortunately, all patients in this series made a full cardiac recovery following immunosuppressive and other therapies, though specific treatment regimens differed.

All received IVIG (duration of therapy varied from 2 to 5 days), and high dose steroids followed by a steroid taper, and patients 1 and 2 also received anakinra. IVIG and steroids have become a mainstay of treatment for MIS-A based on management guidelines for MIS-C and Kawasaki Disease, which share clinical and immunologic features (2). Case reports of success following targeted immunologic therapy are documented for both MIS-A and MIS-C. The most commonly employed biologic immunomodulators are anakinra (7, 8), an IL-1 receptor antagonist, and tocilizumab (7), an IL-6 receptor antagonist, often in conjunction with IVIG and steroids, though there is not yet clinical trial or biological mechanistic data to support this. In the aforementioned systematic review, 60.2% of MIS-A patients received steroids, 37.2% received IVIG and 10.2% received biologics such as anakinra.

Case 1 failed to improve on IVIG and steroids alone, and thus anakinra was added, after which he also made a full recovery. Anakinra, IVIG, and steroids were given simultaneously with therapies for sepsis and DKA in Case 2, with subsequent full cardiac recovery. Case 3 did not receive anakinra but also made a full cardiac recovery, suggesting that steroids and/or IVIG may be beneficial in overlap cases which share features of MIS-A and SARS-CoV-2 viral myocarditis. No adverse events were attributed to anakinra in these three cases, supporting the conclusions of prior case reports (7, 8) that anakinra is a safe and effective treatment option in MIS-A unresponsive to IVIG and steroids alone, or as part of initial treatment in severe cases.

As has been described for MIS-C (9, 10), all three MIS-A cases were characterized by elevated inflammatory biomarkers including CRP, ESR, procalcitonin and IL-6. The additional longitudinal immunoprofiling performed for Case 1 demonstrated that elevated IL-8, IL-18, TNFR-1, MMP-8, and other inflammatory cytokines may characterize MIS-A, with normalization correlating with recovery. Our findings are consistent with a recent study (11) that evaluated inflammatory markers in three MIS-A cases at a single timepoint and also found elevated levels of IL-6, IL-10, IP-10, and IL-18 and ICAM-1. TNFR-1, Ang2, ICAM-1, SP-D, and MMP-8 were not measured in this study, and no convalescent or healthy controls were used for reference. A deep immunoprofiling study (12) of a single MIS-A patient also observed elevated levels of IL-10 and IP-10 compared to convalescent controls, but did not find increases in the other inflammatory proteins that were elevated in case 1.

A recent retrospective analysis (13) compared 25 patients with MIS-A to 13 with COVID-19-related myocarditis that did not meet the U.S. CDC MIS-A criteria (5). They found that MIS-A patients had higher IL-22, IL-17, and TNF- α, and lower interferon- α2 and IL-8 levels. In addition, they detected RNA polymerase III autoantibodies in 54% of MIS-A-negative myocarditis cases. Taken together, these studies highlight that further work is needed to both understand the immunological mechanisms of MIS-A and identify optimal therapies.

Case 2 demonstrates that MIS-A may co-present with new-onset Type 1 diabetes and DKA. Small case series have described a potential association between MIS-C and new onset Type 1 diabetes in children, though the pathophysiology underlying this association is still under investigation (14, 15). Viral infections have long been implicated as triggers for new onset Type 1 diabetes (16, 17), although mechanisms have remained elusive. COVID-19, independent of MIS-A, is a risk factor for new-onset Type-1 diabetes in children (18, 19) and adults (20), but is unclear if MIS-C or MIS-A further increases the risk for disease presentation. Whether the proinflammatory state of MIS-A/C may unmask Type 1 diabetes in those with pre-existing pancreatic islet cell autoantibodies needs to be further investigated. Nonetheless, clinicians should consider MIS-A in a patient with first time DKA following a SARS-CoV-2 infection.

Case 3 had the greatest diagnostic uncertainty as the patient had the shortest interval between prodromal illness and onset of cardiac disease (10 days) and did not have laboratory or clinical evidence of COVID-19 prior to hospitalization. The presumptive diagnosis of MIS-A was made after the extensive in-hospital workup for viral and inflammatory myocarditis returned negative. This case illustrates the overlapping features of MIS-A and COVID-19 myocarditis (13, 21), a heterogeneous condition that includes direct myocardial infection, autoimmune or inflammatory etiologies, and stress-induced (Takotsubo) pathology (21, 22).

## Conclusion

These cases demonstrate that MIS-A, as defined by the US CDC (5), is a heterogeneous syndrome with diverse clinical features that can co-occur with other acute illnesses. While MIS-A is a life-threatening disease, with timely recognition and access to advanced cardiac support and immunomodulatory therapies, clinical recovery is achievable. Clinicians should consider MIS-A in young adults who presents with new onset heart failure or unexplained shock, and a history of recent confirmed or clinically suspected SARS-CoV-2 infection. MIS-A shares clinical and immunologic features with MIS-C, and the published treatment guidelines for MIS-C may represent a reasonable treatment approach for patients with MIS-A. The realization that Case 3 had detectable SARS-CoV-2 viral RNA in cardiac tissue highlights the overlapping clinical features of MIS-A with COVID-19 associated myocarditis. Co-occurrence of Type 1 diabetes with MIS-A suggests that autoimmunity may underpin susceptibility to MIS-A and raises the question of whether autoantibody cross-reactivity drives myocardial damage in some patients.

Together, these cases broaden our understanding of the clinical features and pathophysiology of MIS-A. Studies that further characterize the inflammatory signatures and autoantibody profiles among patients with MIS-A and MIS-C are needed to elucidate the cause of these syndromes and to guide optimal treatment.

## Data availability statement

The raw data supporting the conclusions of this article will be made available by the authors, without undue reservation.

## Ethics statement

Written informed consent was obtained from the individual(s) for the publication of any potentially identifiable images or data included in this article.

## Author contributions

KB, SF, CB, CL, and SP: study conception and design. KB, NS, CH, CC, MM, SF, AC, CB, CL, and SP: data collection. KB, NS, CH, CC, MM, SF, AC, SH, KV, CB, CL, and SP: analysis and interpretation of results. KB, NS, CC, LS, CL, and SP: draft manuscript preparation. All authors reviewed the results and approved the final version of the manuscript.

## Funding

This study was performed with support from the NIH (NCCIH National Center for Complementary and Integrative Health K23AT011768), the National Institute of Allergy and Infectious Diseases-sponsored Immunophenotyping Assessment in a COVID-19 Cohort (IMPACC) Network (NIH/NIAID U19 AI1077439), as well as from the National Heart, Lung and Blood Institute [NHLBI R35 HL140026 (CC) and K23HL138461-01A1 (CL)], and the Chan Zuckerberg Biohub (CL).

## Conflict of interest

The authors declare that the research was conducted in the absence of any commercial or financial relationships that could be construed as a potential conflict of interest.

## Publisher’s note

All claims expressed in this article are solely those of the authors and do not necessarily represent those of their affiliated organizations, or those of the publisher, the editors and the reviewers. Any product that may be evaluated in this article, or claim that may be made by its manufacturer, is not guaranteed or endorsed by the publisher.
