# Supplementary material for: Clinical and biological heterogeneity of multisystem inflammatory syndrome in adults following SARS-CoV-2 infection: a case series
Source: Front Med (Lausanne). 2023 Jul 6;10:1187420. doi: 10.3389/fmed.2023.1187420 (PMC10357379; doi:10.3389/fmed.2023.1187420)
Supplement: Supplementary file 1 [file Table_1.pdf]

Supplemental Table 1: Clinical Findings on Admission and Key Laboratory Values

	PATIENT 1	PATIENT 2	PATIENT 3	
**SIGNS/SYMPTOMS**				
FEVER/CHILLS	+	Hypothermia at admission, fever hospital day 1	+	
DYPSNEA	+	unable to assess	+	
CHEST PAIN	+	unable to assess	-	
COUGH	+	unable to assess	-	
GI SYMPTOMS	+	+	+	
CONJUNCTIVITIS	+	-	+	
RASH	+	-	-	
ALTERED MENTAL STATUS	-	+	-	
**GENERAL LABORATORY FINDINGS**				Normal values
WBC ($X10^9/L$)	25.9	12.7*	31.8	3.4 - 10.0 x10E9/L
PLATELETS ($X10^9/L$)	87	243*	241	140 - 450 x10E9/L
CREATININE (MG/DL)	2.08	3.69	1.62	0.73 - 1.24
GLUCOSE (MG/DL)	382	1,959	131	70 - 199
LACATE (MMOL/L)	10.9	4.8	2.1	0.5 - 2.0
TROPONIN (µG/L)	4.24	22.57	o	< 0.02
HIGH SENSITIVITY TROPONIN (NG/L)	o	o	5,713	<= 45
BNP (PG/ML)	1618	300	o	< 25
NT-PRO-BNP (PG/DL)	o	o	34,645	< 125
ESR (MM/H)	73	13	91	< 10
CRP (MG/L)	443	232	326.7	< 5.1
PROCALCITONIN (µG/L)	13.53	22.0	18.08	< 0.26
FERRITIN (µG/L)	5,056	5,645	8,219	48 - 420
IL-6 (PG/ML)	27.8	21	o	< 5.0
URINE TOXICOLOGY	negative	negative	positive for oxycodone	
**RHEUMATOLOGIC LABORATORY FINDINGS**				
ANA	-	o	-	
DSDNA	-	o	-	
SMITH	o	o	-	
RNP	o	o	-	
C3/C4	o	o	-	
SSA/SSB	o	o	-	
ANTI CENTROMERE	o	o	-	
SCL-70	o	o	-	
RHEUMATOID FACTOR	-	o	o	
ANCA	-	o	-	
**INFECTOUS DISEASE LABORATORY FINDINGS**				
SARS-COV-2 PCR	-	+, Ct 36.1	+, Ct 34.7	
SARS-COV-2 ANTIGEN	o	+	o	
SARS-COV-2 IGG ANTIBODY	+	+	+	
BLOOD CULTURES	negative	*S. marcescens, S. aureus*	negative	
HIV	-	-	-	
CMV IGM/IGG	-	o	-	
EBV IGM/IGG	-	o	+	
COXSACKIE VIRUS AB	o	o	+	
HSV	o	o	+	
GROUP A STREP	o	o	-	
BORELLIA BURGDORFERI	o	o	-	
ADENOVIRUS	o	o	-	
RICKETTSIA AB	o	o	-	
MYCOPLASMA IGG	o	o	-	
BARTONELLA AB	o	o	-	
LEGIONELLA AB	o	o	-	
TRYPANOSOME AB	o	o	-	
RESPIRATORY VIRAL PANEL**	-	o	-	
AFB CULTURES	o	o	-	
PARVOVIRUS PCR	-	o	-	
ENTEROVIRUS PCR	-	o	-	
**CARDIOLOGY STUDIES**				

	Patient 1	Patient 2	Patient 3
CARDIAC CATHETERIZATION	not done	no significant coronary artery disease	no significant coronary artery disease
TRANSTHORACIC ECHOCARDIOGRAM			

CASE 1:
1. LV FUNCTION IS SEVERELY DECREASED. LV EJECTION FRACTION IS ESTIMATED TO BE 20 - 25%. THERE IS GLOBAL HYPOKINESIS. THERE IS NO EVIDENCE OF LV THROMBUS. THERE IS AN IMPELLA DEVICE IN THE LV.
2. THE RIGHT VENTRICULAR VOLUME IS MILDLY INCREASED. RIGHT VENTRICULAR FUNCTION IS MODERATELY DECREASED.
3. LEFT ATRIAL SIZE IS NORMAL. THERE IS MILD RIGHT ATRIAL ENLARGEMENT.
4. NO HEMODYNAMICALLY SIGNIFICANT VALVULAR DISEASE.
5. DIASTOLIC FUNCTION IS INDETERMINATE.
6. THE RV SYSTOLIC PRESSURE IS AT LEAST 37 MMHG BASED ON A RIGHT ATRIAL PRESSURE OF 15 MMHG.
7. NO PERICARDIAL EFFUSION NOTED. THE INFERIOR VENA CAVA IS GREATER THAN 21 MM IN DIAMETER AND COLLAPSES LESS THAN 50% WITH INSPIRATION CONSISTENT WITH A RIGHT ATRIAL PRESSURE OF 15 MMHG.
8. Aortic root dimension is normal.

Case 2:
1. THE LEFT VENTRICULAR VOLUME IS NORMAL. LV FUNCTION IS SEVERELY DECREASED. THERE IS AN IMPELLA DEVICE IN THE LV.
2. THE RIGHT VENTRICULAR VOLUME IS NORMAL. RIGHT VENTRICULAR FUNCTION IS MODERATELY DECREASED.
3. LEFT ATRIAL SIZE IS NORMAL. RIGHT ATRIAL SIZE IS NORMAL.
4. THERE IS NO HEMODYNAMICALLY SIGNIFICANT VALVULAR DISEASE.
5. DIASTOLIC FUNCTION WAS NOT ASSESSED ON THIS STUDY.
6. THE PULMONARY ARTERY SYSTOLIC PRESSURE IS AT LEAST 12 MMHG PLUS THE RA PRESSURE.
7. NO PERICARDIAL EFFUSION NOTED. MECHANICAL VENTILATION PRECLUDES ACCURATE ESTIMATION OF RIGHT ATRIAL PRESSURE.
8. Aortic root dimension is normal.

Case 3
1. THE LEFT VENTRICULAR VOLUME IS MILDLY INCREASED. LV FUNCTION IS NORMAL. LV EJECTION FRACTION IS ESTIMATED TO BE 55 TO 60%. THERE IS MODERATE LEFT VENTRICULAR HYPERTROPHY. THE PATTERN OF LEFT VENTRICULAR HYPERTROPHY IS ECCENTRIC.
2. THE RIGHT VENTRICULAR VOLUME IS MILDLY INCREASED. RIGHT VENTRICULAR FUNCTION IS NORMAL.
3. LEFT ATRIAL SIZE IS NORMAL. THERE IS MILD RIGHT ATRIAL ENLARGEMENT.
4. THERE IS NO HEMODYNAMICALLY SIGNIFICANT VALVULAR DISEASE.
5. TACHYCARDIA PRECLUDES THE ACCURATE EVALUATION OF DIASTOLIC FUNCTION.
6. THE PULMONARY ARTERY SYSTOLIC PRESSURE IS AT LEAST 15 MMHG PLUS THE RA PRESSURE.
7. NO PERICARDIAL EFFUSION NOTED. MECHANICAL VENTILATION PRECLUDES ACCURATE ESTIMATION OF RIGHT ATRIAL PRESSURE.
8. Aortic root dimension is normal.

*Patient 3 was profoundly hypovolemic on admission
**Respiratory viral panel includes: Influenza A (H1 and H3) and B, RSV A, B, Parainfluenza 1 – 3, Rhinovirus, Metapneumovirus, and Adenovirus
o = no result
LVEF/RVEF = left/right ventricular ejection fraction
Table includes the highest values reported during admission.
