# Supplementary material for: Clinical and biological heterogeneity of multisystem inflammatory syndrome in adults following SARS-CoV-2 infection: a case series
Source: Front Med (Lausanne). 2023 Jul 6;10:1187420. doi: 10.3389/fmed.2023.1187420 (PMC10357379; doi:10.3389/fmed.2023.1187420)
Supplement: Supplementary file 2 [file Table_2.pdf]

Supplemental Table 2: Clinical and Demographic Characteristics of Non-MIS-A Control Patients 3 Months Following COVID-19 Diagnosis

**Age (average, range)**	**52 (32 - 75)**
**Male sex**	16 (76%)
**BMI (average, range)**	30 (22041)
**Race**	
**White**	3 (14%)
**Black / African American**	1 (5%)
**American Indian / Alaska Native**	1 (5%)
**Asian**	1 (5%)
**Other / Multiple Races**	14 (67%)
**Ethnicity**	
**Hispanic/Latino Ethnicity**	15 (71%)
**Outcomes**	
**Corticosteroid treatment**	10 (48%)
**Vaccination**	0 (0%)
**Outcomes**	
**Floor admission**	17 (81%)
**ICU admission**	4 (19%)
**Mechanical ventilation**	2 (10%)
**Mortality**	0 (0%)
